# Prevalence of multimorbidity and uptake of guideline-directed medicines for cardiovascular conditions in Australian hospitalised adults: a cross-sectional study

**DOI:** 10.1136/bmjopen-2025-103243

**Published:** 2026-02-10

**Authors:** Joshua M Inglis, Gillian E Caughey, Danny Liew, Sepehr Shakib

**Affiliations:** 1 School of Pharmacy and Biomedical Sciences, College of Health, Adelaide University, Adelaide, South Australia, Australia; 2 Department of Clinical Pharmacology, Flinders Medical Centre and Flinders University, Adelaide, South Australia, Australia; 3 Registry of Senior Australians Research Centre, South Australian Health and Medical Research Institute, Adelaide, South Australia, Australia; 4 Registry of Senior Australians Research Centre, Caring Futures Institute, College of Nursing and Health Sciences, Flinders University, Adelaide, South Australia, Australia; 5 School of Medicine, College of Health, Adelaide University, Adelaide, South Australia, Australia; 6 Faculty of Health, Medicine and Behavioural Sciences, The University of Queensland, Brisbane, Queensland, Australia; 7 Northern Adelaide Local Health Network, Adelaide, South Australia, Australia

**Keywords:** Multimorbidity, Frailty, Polypharmacy, Drug Utilization, Cardiovascular Disease

## Abstract

**Abstract:**

**Objectives:**

Multimorbidity, defined as two or more chronic medical conditions, leads to the use of multiple medicines, including for cardiovascular conditions. This is associated with frailty and an increased risk of medication-related harm. Hospitalised adults have higher rates of multimorbidity and frailty compared with non-hospitalised adults. The aim of this study was to examine the use of medicines for hypertension, ischaemic heart disease and atrial fibrillation among patients with multimorbidity and frailty, who are generally not well represented in clinical trials.

**Design:**

A cross-sectional study was performed of adults aged ≥45 years with inpatient admissions during an 18-month period. Regular medications prescribed at discharge and coding data were obtained from the electronic medical record and hospital datasets.

**Primary and secondary outcome measures:**

The prevalence of multimorbidity (using coded chronic medical conditions or the RxRisk pharmaceutical comorbidity index), frailty (using hospital frailty risk score) and polypharmacy (defined as ≥5 medicines) were calculated. The uptake of medicines recommended by the Australian Therapeutic Guidelines for patients with coded hypertension, ischaemic heart disease and atrial fibrillation was also assessed.

**Setting:**

Two large acute care, teaching hospitals in Adelaide, South Australia.

**Participants:**

23 980 unique patients were identified.

**Results:**

69% (n=16 637) of patients had multimorbidity using the coding definition compared with 94% (n=22 620) using the pharmaceutical comorbidity score. 81% (n=19 366) had polypharmacy and 46% (n=11 091) had frailty. More than 85% of patients with hypertension were taking an antihypertensive. More than 75% of patients with ischaemic heart disease were taking an antithrombotic or a lipid-lowering agent and more than 50% were taking an agent acting on the renin-angiotensin system. Over 70% of patients with atrial fibrillation without a contraindication to anticoagulation were taking an anticoagulant. Patients with multimorbidity were 11–51% more likely to be taking an antihypertensive, antithrombotic or lipid-lowering medicine for the respective cardiovascular conditions, whereas those with frailty were 31–48% less likely to be taking guideline-directed medicines for all conditions studied.

**Conclusions:**

Over two-thirds of hospitalised patients with these cardiovascular conditions were taking at least one guideline-directed medicine. Medication use was generally more common in multimorbidity and less common in frailty. Outcomes studies are needed to quantify the risks and benefits of cardiovascular medicines in these patients.

**STRENGTHS AND LIMITATIONS OF THIS STUDY**

- Large population of hospital inpatients reflecting real-world practices and use of coding data to determine the presence of cardiovascular conditions.
- Medication orders at the time of hospital discharge may not equate to long-term medication use.
- Inability to include as needed medications in the count of medications may have underestimated the rates of polypharmacy.
- Use of International Classification of Diseases, 10th Revision (ICD-10) codes from hospital coding data may be insensitive for the detection of select chronic medical conditions.
- Multimorbidity and frailty were both assessed using ICD-10 codes and the link between these is acknowledged.

## Introduction

 Multimorbidity, defined as the presence of two or more chronic medical conditions, is common and reported in up to 44% of hospitalised adults in Australia.^1^ The presence of multimorbidity is associated with adverse health outcomes, including increased mortality and reduced quality of life.^2 3^ The occurrence of multimorbidity increases with age and as a result, occurs mostly in middle-aged and older adults.^4^

Multimorbidity frequently coexists with frailty, a health state characterised by increased vulnerability to external stressors.^5 6^ Multimorbidity is also associated with polypharmacy, the use of five or more medicines, often due to the prescription of multiple guideline-directed medicines for chronic diseases.^7^ However, there is uncertainty in the risks and benefits of these medications in this population, as patients with multimorbidity and frailty are generally not well represented in clinical trials.^8^,^12^ Hospitalised patients have higher rates of multimorbidity, polypharmacy and frailty compared with non-hospitalised adults, which may place them at increased risk of medication-related harms.^113^,^16^ A recent Cochrane review demonstrated that medication reviews during hospitalisation can optimise medication use and likely reduce hospital readmissions and emergency department presentations.^17^

The biggest contributor to multimorbidity burden in hospitalised adults is cardiovascular conditions. Almost half of hospitalised adults have hypertension and one-quarter have heart disease, with over 70% of these patients having multimorbidity.^1^ Hypertension is the leading global modifiable risk factor for cardiovascular morbidity and mortality, while ischaemic heart disease is the highest global ranked cause of cardiovascular deaths.^18^ Atrial fibrillation prevalence increases with age and leads to significant morbidity and mortality.^18^ Polypharmacy is common in cardiovascular conditions with use of multiple medications to control risk factors and prevent cardiovascular events such as myocardial infarction, stroke and heart failure. Cardiovascular medicines account for one-third of total prescription medication dispensed in Australia and use increases with age.^19^ Two-thirds of older adults in Australia were dispensed two or more cardiovascular medicines over a 6-month period in 2019, while 17% were dispensed five or more cardiovascular medicines.^20^

To the best of our knowledge, there are no contemporaneous studies in hospitalised Australian adults assessing the prevalence of multimorbidity, polypharmacy and frailty. Additionally, given their high burden to the hospitalised population, and clear treatment targets and prescribing guidelines, the factors associated with the use of guideline-directed medicines for these conditions will be examined. Understanding the influence of multimorbidity and frailty on inpatient prescribing practices or medical tolerance will identify potential targets to optimise medication use to reduce harms.^21^ The aim of this study was therefore to examine the use of medicines for hypertension, ischaemic heart disease and atrial fibrillation among patients with multimorbidity and frailty, who are generally not well represented in clinical trials.

## Method

### Study type and setting

A cross-sectional study reported in accordance with the REporting of studies Conducted using Observational Routinely-collected health Data statement^22^ was conducted using hospital admission data from the Royal Adelaide Hospital and the Queen Elizabeth Hospital in Adelaide, South Australia, between January 2022 and June 2023. The Royal Adelaide Hospital has approximately 95 000 inpatient admissions annually, while the Queen Elizabeth Hospital has approximately 40 000.^23^ These hospitals use Sunrise electronic medical record (EMR), which is a modification of the Altera Digital Health Sunrise product via the Sunrise Clinical Performance Module (Altera Digital Health, Niagara Falls, New York). There was no data linkage. Given all first hospitalisations during the study period were included, no further sampling strategy was applied.

### Participants

The inclusion criteria were all adults aged ≥45 years with first admissions via the emergency department under specific units who manage patients with general comorbidities (see online supplemental appendix A), such as general medicine, general surgery and geriatrics. These broad prespecified inclusion criteria were applied to avoid selection bias through the overrepresentation of frequently hospitalised individuals. Patients were required to have a length of in-patient stay greater than 48 hours to allow sufficient time for the treating clinicians to chart their preadmission medications into the EMR. Patients with palliative care consultations who did not survive to discharge were excluded. It is acknowledged that there will be selection bias introduced by the minimum length of stay, exclusion of patients who did not survive to discharge and exclusion of patients admitted under highly specialised units. However, this was necessary to achieve the study aims while ensuring that the cohort remained representative of the general inpatient population.

### Study variables

The study variables were age, gender, admitting unit, chronic medical conditions, medication orders at the time of discharge, serum creatinine on admission and the presence of hepatic impairment. Age, gender, medication orders at the time of discharge, serum creatinine on admission were extracted from the EMR. Medicine orders were mapped to the WHO Anatomical Therapeutic Chemical (ATC) classification system.^24^ Regular medications were defined as medication orders in the EMR at the time of discharge excluding short-term and as needed medications. Prophylactic orders for enoxaparin were removed along with all intravenous, subcutaneous and intramuscular medicines other than those for long-term use (eg, insulins, antiresorptives, glucagon-like peptide-1 receptor agonists and erythrocyte stimulating agents) as well as anti-infectives (ATC Code J). Polypharmacy was defined as five or more long-term medicines classified using ATC codes in accordance with the accepted definition.^7^ Hyperpolypharmacy was defined as 10 or more long-term medications. The Rx-Risk V comorbidity index based on medication use was calculated.^25^ Renal function was assessed using the serum creatinine on admission.

Hospital coding data using the International Classification of Diseases, 10th Revision (ICD-10)-Australian Modification was extracted for the identified admission. These contain supplementary codes to record comorbidities.^26^ Multimorbidity was identified using a published count of 40 chronic medical conditions from hospital coding data.^27^ Individuals with two or more coded chronic medical conditions during the hospital admission were considered to have multimorbidity in accordance with the previous study.^27^ This definition was chosen as the most widely used and internationally adopted method of identifying multimorbidity from administrative data. It is one of the recommended methods when measuring prevalence and had published ICD-10 code lists when this study was devised.^28^ Given the potential for undercoding of chronic medical conditions in administrative data, a sensitivity analysis was performed by identifying multimorbidity using the Rx-Risk V comorbidity index based on medications orders. This was performed to mitigate potential misclassification bias through the use of coding data. The individual chronic conditions were identified from the multimorbidity count using ICD-10 codes to show the frequency of each condition. The comorbidities of interest (hypertension, ischaemic heart disease and atrial fibrillation) were also identified using select ICD-10 codes (see online supplemental appendix B). The hospital frailty risk score, which uses ICD-10 codes for markers of frailty (eg, cognitive impairment, functional dependence, falls and fractures), was used to identify those with frailty.^29^ Individuals with an intermediate or high risk of frailty using this score were considered to be frail, consistent with the derivation study. Hepatic impairment was also identified using ICD-10 codes from this algorithm (online supplemental appendix C).^30^

Use of guideline-recommended medicines for hypertension, ischaemic heart disease and atrial fibrillation was identified using the Australian Therapeutic Guidelines which are an independent, evidence-based prescribing resource (see online supplemental appendix D).^31^ Antihypertensives included ACE inhibitor (C09A, C09B), angiotensin receptor blocker (C09C, C09D), dihydropyridine calcium channel blockers (C08CA) and thiazide or thiazide-like diuretics (C03A, C03BA). For ischaemic heart disease, this included any antiplatelet or anticoagulant, lipid-lowering agents and agents acting on the renin-angiotensin system as previously defined. Antiplatelets included aspirin (B01AC06, B01AC56), clopidogrel (B01AC04) and ticagrelor (B01AC24). Anticoagulants included apixaban (B01AF02), rivaroxaban (B01AF01), dabigatran (B01AE07), warfarin (B01AA) and enoxaparin (B01AB05). Lipid-lowering agents included statins (C10AA, C10BX), ezetimibe (C10AX09) and proprotein convertase subtilisin/kexin type 9 (PCSK9) inhibitors (C10AX13, C10AX14). For the uptake of anticoagulation in patients with atrial fibrillation, those with contraindications to anticoagulation based on a published algorithm were excluded (see online supplemental appendix E).^32^ These included patients with histories of intracranial haemorrhage, intracranial masses, end-stage liver disease, previous gastrointestinal haemorrhage and blood dyscrasias.

### Statistical analysis

Descriptive statistics were calculated for baseline characteristics of the study cohort, including age, gender, admitting unit, number of regular medicines, as well as prevalence of multimorbidity, polypharmacy and frailty.

Each of the cardiovascular conditions was stratified based on the presence or absence of multimorbidity and frailty in individuals with the condition. The prevalence of guideline-directed medication use for individuals with hypertension, ischaemic heart disease and atrial fibrillation is expressed as a percentage (with upper and lower bounds of the 95% CI) of the total patients with each cardiovascular condition and stratified by presence of multimorbidity or frailty.

The characteristics of patients with cardiovascular conditions of interest were compared for those taking and not taking each class of guideline-directed medicine. This was using the Wilcoxon rank-sum test, Pearson’s χ² test or Fisher’s exact test as appropriate. Multivariate logistic regression was used to determine the association between multimorbidity and frailty with the use of guideline-directed medicines. The confounding factors of age, gender, number of medications, renal function and the presence of chronic liver disease were chosen as these clinically influence medication use. Older age is associated with an increased risk of cardiovascular events and medication-related harm. Gender was chosen to account for sex-based differences in medication use. The number of medications was chosen to represent baseline medication burden which may influence prescribing practices through risk of drug–drug interactions and resulting caution in prescribing. Renal and hepatic function represents the primary modes of drug excretion and influences medication use. Renal function was assessed using the serum creatinine on admission and hepatic function using select ICD-10 codes (see online supplemental appendix C). The other potential confounders were socioeconomic status, ethnicity and inpatient blood pressure readings for hypertension treatment. Socioeconomic status and ethnicity were not available in our dataset. Blood pressure readings were not included in the analysis given that these are a dynamic vital sign and that blood pressure targets for hypertension are based on outpatient rather than inpatient measurements. Changes in prescribing patterns over time were not considered a confounder given the study was undertaken over a defined 18 months period and these medications had all been available and recommended by guidelines for several years prior. Given this, the confounders adjusted for in the logistic regression were age, gender, number of regular medicines, the logarithm of serum creatinine on admission and hepatic impairment. Missing data were minimal (<5%) due to the structured nature of the EMR. These were limited to gender and serum creatinine. Individuals with missing values were excluded from the regression analyses. Given the small proportion of missing data, imputation was not performed. This is not expected to have influenced the outcomes. Variables were assessed for collinearity using the variance inflation factor (VIF). All VIFs were less than 2 indicating that there was no significant collinearity between the covariates. No other sensitivity analyses were performed, as results were expected to be robust given the low proportion of missing data and inclusion of all eligible hospitalisations. Analysis was performed using RStudio, V.4.1.2 (R Foundation for Statistical Computing, Vienna, Austria) by one author with access to the dataset (JMI). Those with p<0.05 were considered statistically significant.

### Patient and public involvement

Consumers were not involved in the design of the study. A consumer with lived experience as a carer of a family member with multimorbidity provided feedback on the results, their interpretation and the implications for patients. Their family member died from inanition 3 weeks after their second neck of femur fracture. The study results will be disseminated to a group of consumers through a series of workshops being conducted for a related research project on medication management at transitions of care following hospital admission.

## Results

### Overall study cohort characteristics

There were 23 980 unique patients with hospital admissions during the study period (table 1). The median age was 74 years (IQR 62–84) with most being older adults (71%, n=16 914) rather than middle-aged (29%, n=7066). 47% of patients were female (n=11 241). Over two-thirds of all patients were admitted under a medical unit (68%, n=16 220) with fewer being admitted under a surgical unit (30%, n=7184) or mental health (2%, n=576). The median number of chronic medical conditions was 4 (IQR 5–7) using the pharmaceutical comorbidity index with 30% (n=7272) of patients having 2–4 conditions and 64% (n=15 348) of patients having 5 or more conditions. 69% of all patients (n=16 637) had multimorbidity using the hospital diagnoses compared with 94% using the pharmaceutical comorbidity index. 81% of all patients (n=19 366) were exposed to polypharmacy and 38% of all patients (n=9103) were exposed to hyperpolypharmacy. The median number of regular medications was 8 (IQR 5–11). 46% of all patients (n=11 091) were frail.

**Table 1:** Baseline characteristics of hospitalised middle-aged and older adults

Characteristic	N=23 980*
Age, median (IQR)	74 (62–84)
Age category, n (%)	
45–64	7066 (29)
65–74	5069 (21)
75–84	6197 (26)
85–94	4877 (20)
Over 95	771 (3.2)
Gender, n (%)	
Female	11 241 (47)
Male	12 738 (53)
Category of admitting unit, n (%)	
Medicine	16 220 (68)
Mental health	576 (2.4)
Surgery	7184 (30)
Number of long-term medications, median (IQR)	8 (5–11)
Number of long-term medicines, n (%)	
1–4	4614 (19)
5–9	10 263 (43)
Greater than 9	9103 (38)
Polypharmacy	
Absent	4614 (19)
Present	19 366 (81)
RxRisk V comorbidity score, median (IQR)	4 (5–7)
0–1	1360 (5.7)
2–4	7272 (30)
5 or more	15 348 (64)
Multimorbidity calculated using RxRisk V	
Absent	1360 (5.7)
Present	22 620 (94)
Number of chronic medical conditions based on hospitalisation diagnoses, median (IQR)	2 (1–3)
Chronic medical conditions based on coding, n (%)	
0	2335 (9.7)
1	5008 (21)
2	6234 (26)
3 or more	10 403 (43)
Multimorbidity calculated using hospitalisation diagnoses, n (%)	
Absent	7343 (31)
Present	16 637 (69)
Frailty, n (%)	11 091 (46)

Missing values (gender=1).

* Median (Q1, Q3); n (%).

### Chronic medical conditions by multimorbidity and frailty status

**Figure 1:**
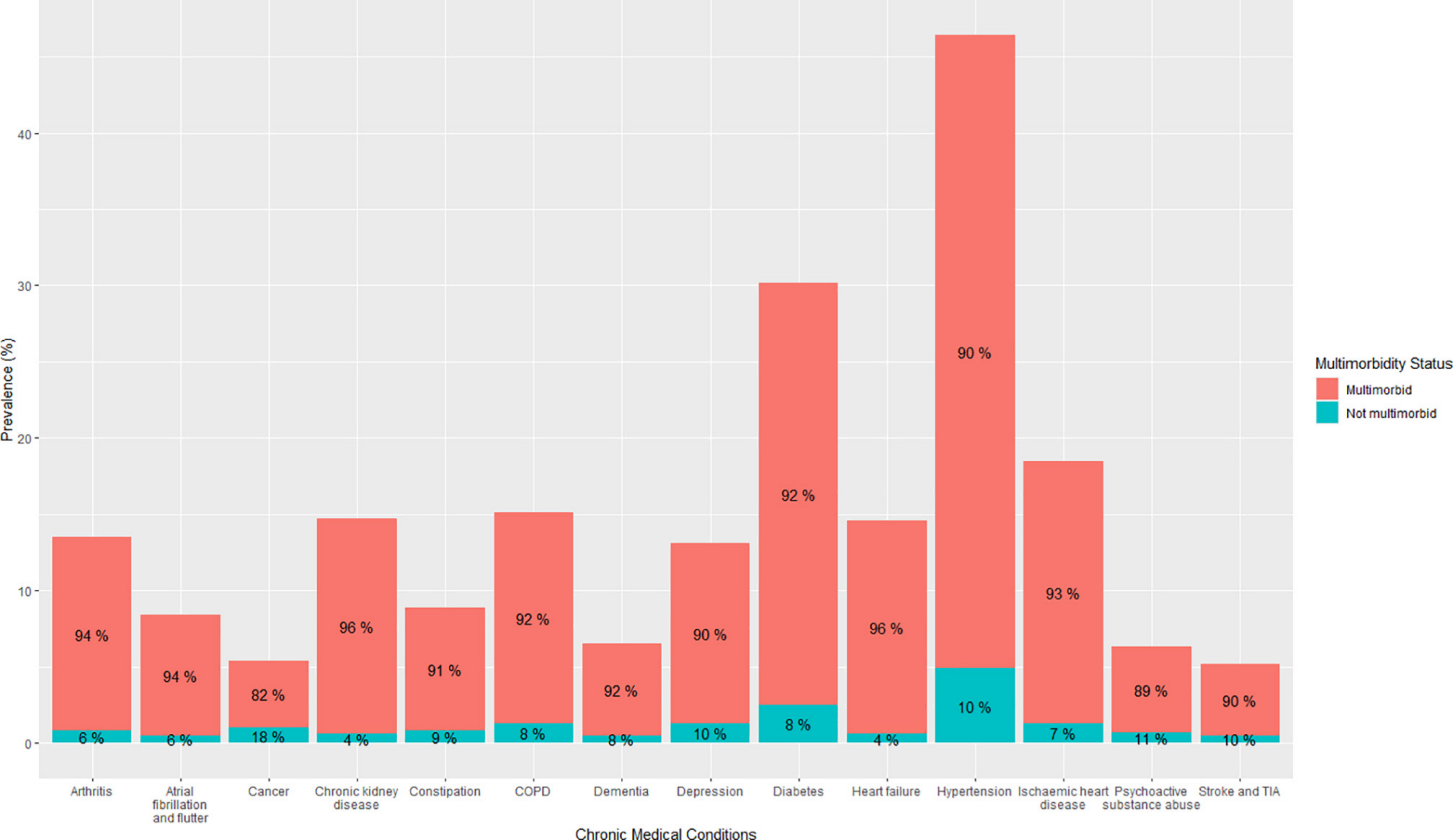
Prevalence of chronic conditions and multimorbidity in hospitalised adults ≥45 years. Percentages within each column are per cent of those with that condition who had multimorbidity. Only those conditions with a prevalence of ≥5% are presented. COPD, chronic obstructive pulmonary disease; TIA, transient ischaemic attack.

**Figure 2:**
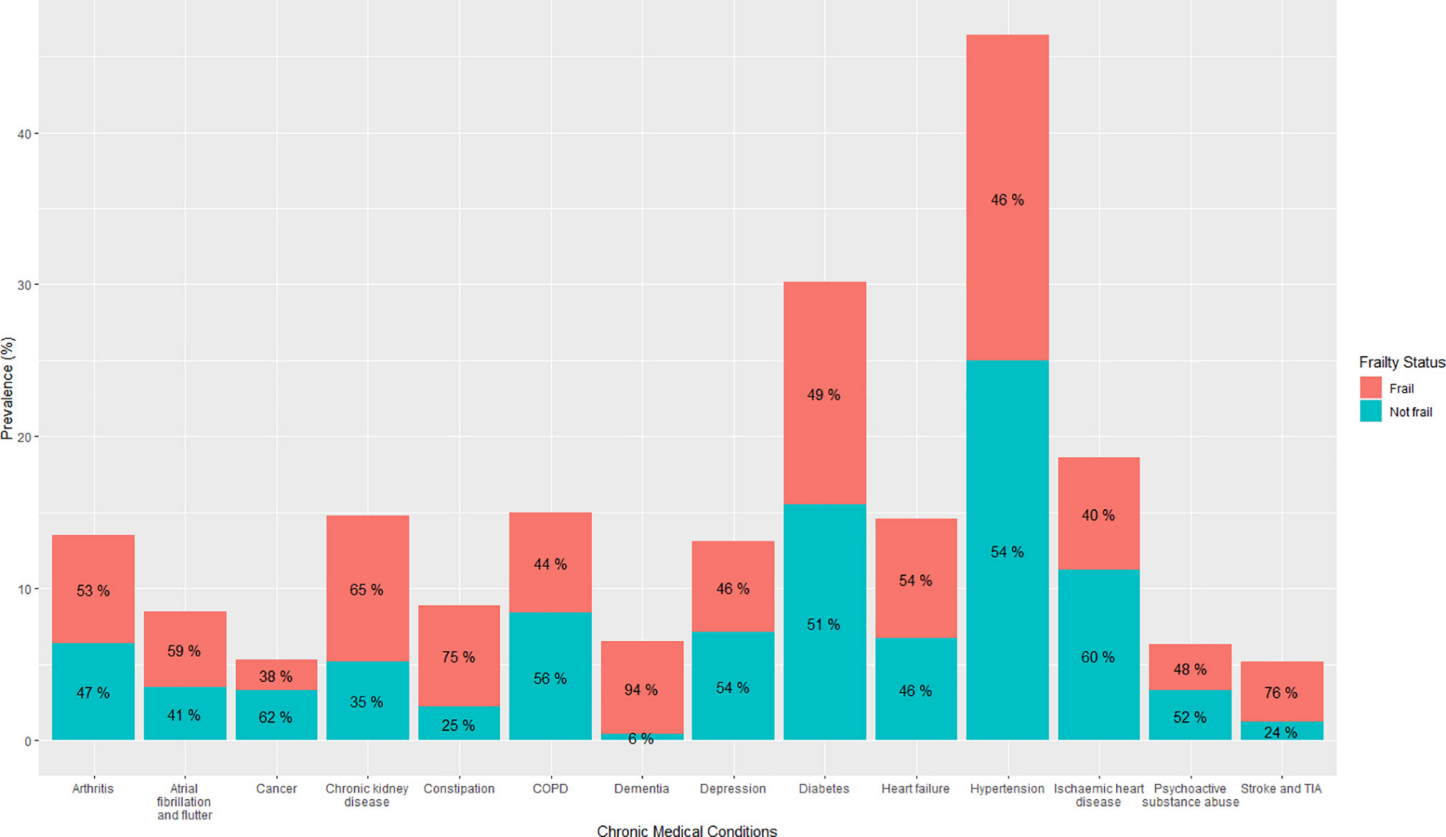
Prevalence of chronic conditions in hospitalised adults ≥45 years in addition to the prevalence of frailty based on coding within each condition. Percentages within each column are per cent of those with that condition who had multimorbidity. Only those conditions with a prevalence of ≥5% are presented. COPD, chronic obstructive pulmonary disease; TIA, transient ischaemic attack.

The most common coded chronic medical conditions during the identified hospital admission in this population were hypertension (46.4%, n=11 122), diabetes (30.2%, n=7250), ischaemic heart disease (18.6%, n=4451), chronic obstructive pulmonary disease (15.0%, n=3602), chronic kidney disease (14.8%, n=3545), heart failure (14.6%, n=3501), arthritis (13.5%, n=3226) and depression (13.0%, n=3128) (figures1  2). The comorbidities with the highest proportion of individuals with multimorbidity were heart failure (96%), chronic kidney disease (96%), arthritis (94%), atrial fibrillation and flutter (94%) and ischaemic heart disease (93%) (figure 1). The rates of frailty were highest in adults with dementia (94%), stroke/transient ischaemic attack (76%), constipation (75%) and chronic kidney disease (65%) (figure 2).

### Uptake of guideline-directed medicines for selected cardiovascular conditions

Of the 11 122 patients with hypertension, 85.2% (95% CI 84.6% to 85.9%, n=9481) were prescribed an antihypertensive agent, which was similar in the subgroups with multimorbidity (85.5%, 95% CI 84.8% to 86.2%, n=8508) and frailty (82.9%, 95% CI 81.9% to 84.0%, n=4247) (see table 2).

**Table 2:** Uptake of guideline-directed medications for cardiovascular conditions in hospitalised adults ≥45 years

Cardiovascular condition	Guideline-directed medications	Overall prevalence % (95% CI) (n/N)	Prevalence in subgroup with multimorbidity % (95% CI) (n/N)	Prevalence in subgroup with frailty % (95% CI) (n/N)
Hypertension	Antihypertensive	85.2 (84.6 to 85.9) (9481/11 122)	85.5 (84.8 to 86.2) (8508/9952)	82.9 (81.9 to 84.0) (4247/5120)
Ischaemic heart disease	Antiplatelet or anticoagulant	87.1 (86.1 to 88.1) (3879/4451)	87.6 (86.6 to 88.6) (3605/4113)	85.1 (83.3 to 86.7) (1501/1764)
	Lipid-lowering agent	77.9 (76.6 to 79.1) (3466/4451)	77.4 (76.0 to 78.6) (3197/4133)	70.1 (67.9 to 72.3) (1237/1764)
	Agent acting on the renin-angiotensin system	58.0 (56.5 to 59.4) (2581/4451)	57.9 (56.4 to 59.5) (2395/4133)	48.7 (46.3 to 51.1) (859/1764)
Atrial fibrillation without a contraindication to anticoagulation*	Anticoagulant	72.0 (70.0 to 74.2) (1277/1770)	72.8 (70.6 to 74.9) (1208/1659)	69.1 (66.1 to 71.9) (699/1012)

* Excluding blood dyscrasias, cirrhosis, hepatic decompensation, intracranial haemorrhage, intracranial mass and prior gastrointestinal haemorrhage (see online supplemental appendix D).

Of the 4451 patients with ischaemic heart disease, 87.1% (95% CI 86.1% to 88.1%, n=3879) were taking an antiplatelet or anticoagulant, which was similar in the subgroup with multimorbidity (87.6%, 95% CI 86.6% to 88.6%, n=3605) and slightly lower in the subgroup with frailty (85.1%, 95% CI 83.3% to 86.7%, n=1501). Of those with ischaemic heart disease, 77.9% (95% CI 76.6% to 79.1%, n=3466) taking a lipid-lowering agent, which was similar in the subgroup with multimorbidity (77.4%, 95% CI 76.0% to 78.6%, n=3197) and lower in the subgroup with frailty (70.1%, 95% CI 67.9% to 72.3%, n=1237). Of those with ischaemic heart disease, 58.0% (95% CI 56.5% to 59.4%, n=2581) were on an agent acting on the renin-angiotensin system, which was similar in the subgroup with multimorbidity (57.9%, 95% CI 56.4% to 59.5%, n=2395) but lower in those with frailty (48.7%, 95% CI 46.3% to 51.1%, n=859).

Of the 1170 patients with atrial fibrillation without a contraindication to anticoagulation, 72.0% (95% CI 70.0% to 74.2%, n=1277) were on any anticoagulant. This was similar in the subgroup with multimorbidity (72.8%, 95% CI 70.6% to 74.9%, n=1208) but lower in those with frailty (69.1%, 95% CI 66.1% to 71.9%, n=699).

### Factors associated with the use of guideline-directed medicines for selected cardiovascular conditions

Patients with hypertension who were on an antihypertensive were more likely to be female, have multimorbidity and be on more regular medicines compared with those not on an antihypertensive (online supplemental table 1). When controlling for covariates in this group, those with multimorbidity were 51% more likely to be on an antihypertensive (adjusted OR 1.51, 95% CI 1.27 to 1.80) and those with frailty were 43% less likely to be on an antihypertensive (adjusted OR 0.57, 95% CI 0.50 to 0.64) (table 3 and online supplemental table 6).

**Table 3:** Guideline-directed medicine use for cardiovascular conditions and associations with multimorbidity or frailty

Cardiovascular condition	Guideline-directed medicine	Association with multimorbidity, adjusted OR (95% CI)*	Association with frailty, adjusted OR (95% CI)*
Hypertension	Antihypertensive	**1.51 (1.27 to 1.80)**	**0.57 (0.50 to 0.64)**
Ischaemic heart disease	Antiplatelet or anticoagulant	**1.49 (1.05 to 2.14)**	**0.69 (0.57 to 0.84)**
	Lipid-lowering agent	**1.89 (1.36 to 2.67)**	**0.52 (0.44 to 0.61)**
	Any agent acting on renin-angiotensin system	0.93 (0.73 to 1.19)	**0.56 (0.50 to 0.64)**
Atrial fibrillation without a contraindication to anticoagulation	Anticoagulant	1.44 (0.95 to 2.17)	**0.66 (0.53 to 0.83)**

Shown in bold are significant associations.

* Adjusted for age, gender, multimorbidity, frailty, number of regular medicines, logarithm of serum creatinine and hepatic impairment.

Patients with ischaemic heart disease who were on an antiplatelet or anticoagulant were younger, more likely to be male, on more regular medicines, less likely to be frail and less likely to have hepatic impairment compared with those not on an antiplatelet or anticoagulant (online supplemental table 2). When controlling for covariates in this group, those with multimorbidity were 49% more likely to be on an antiplatelet or anticoagulant (adjusted OR 1.49, 95% CI 1.05 to 2.14) and those with frailty were 31% less likely to be on an antiplatelet or anticoagulant (adjusted OR 0.69, 95% CI 0.57 to 0.84) (table 3 and online supplemental table 7).

Patients with ischaemic heart disease who were on a lipid-lowering agent were younger and were less likely to have frailty compared with those not on a lipid-lowering agent (online supplemental table 3). When controlling for covariates in this group, those with multimorbidity were 89% more likely to be on a lipid-lowering agent (adjusted OR 1.89, 95% CI 1.36 to 2.67) and those with frailty were 48% less likely to be on a lipid-lowering agent (adjusted OR 0.52, 95% CI 0.44 to 0.61) (table 3 and online supplemental table 8).

Patients with ischaemic heart disease who were on an agent acting on the renin-angiotensin system were younger, more likely to be male, less likely to be frail and had a lower serum creatinine compared with those not on these agents (online supplemental table 4). When controlling for covariates in this group, there was no association with multimorbidity (adjusted OR 0.93, 95% CI 0.73 to 1.19) and those with frailty were 44% less likely to be on agent acting on the renin-angiotensin system (adjusted OR 0.56, 95% CI 0.50 to 0.64) (table 3 and online supplemental table 9).

In patients with atrial fibrillation without a contraindication to anticoagulation, those prescribed an anticoagulant were more likely to have multimorbidity and less likely to have frailty compared with those not on an anticoagulant (online supplemental table 5). When controlling for covariates, there was no association with multimorbidity (adjusted OR 1.44, 95% CI 0.95 to 2.17) and those with frailty were 34% less likely to be on an anticoagulant (adjusted OR 0.66, 95% CI 0.53 to 0.83) (table 3 and online supplemental table 10).

## Discussion

Multimorbidity is prevalent in hospitalised adults and there were high rates of guideline-recommended medication use for the selected cardiovascular conditions. More than 85% of patients with hypertension were taking an antihypertensive. More than 75% of patients with ischaemic heart disease were taking an antithrombotic or a lipid-lowering agent and more than 50% were taking an agent acting on the renin-angiotensin system. Over 70% of patients with atrial fibrillation without a contraindication to anticoagulation were taking an anticoagulant. Patients with multimorbidity were 11–51% more likely to be taking an antihypertensive, antithrombotic or lipid-lowering medicine for the respective cardiovascular conditions, whereas those with frailty were 31–48% less likely to be taking guideline-directed medicines for all conditions studied. While older adults with multimorbidity and frailty are generally not well represented in clinical trials and uncertainty exists with regard to effectiveness in this population, our findings suggest that clinicians are applying the guideline recommendations for treatment to these patients.^10^,^1233^

The rates of multimorbidity in this hospitalised population (range 69–94%) were higher than previous estimates in the Australian or international setting. The RxRisk comorbidity score has been shown to have fair agreement with self-reported multimorbidity and is likely to be more sensitive than hospital coding data in the Australian setting.^1^ The prevalence of multimorbidity in hospitalised patients is known to be higher than community-dwelling adults, where only 36% were multimorbid in a recent global study.^34^ In comparison, multimorbidity was present in 26% of adults attending general practices and 44% of middle-aged and older adult inpatients in the Australian setting.^1 13^ The prevalence of multimorbidity has been increasing globally in parallel with the ageing population.^34^ The higher rates of multimorbidity in our study compared with previous studies of inpatients may reflect an older hospital population and changes in hospital coding practices. New hospital coding rules were introduced in 2015 that included the use of supplementary codes for comorbidities. Other studies have shown that this led to a significant increase in the coding of hospital comorbidities and may account for the higher rate of multimorbidity in this study.^35^ Furthermore, the restriction to admitting units that manage general comorbidities is likely to have resulted in the inclusion of patient groups with higher rates of multimorbidity leading to higher estimates than previous studies. The breakdown of chronic conditions in this study was broadly consistent with the previous Australian literature.^1 4 36^

The rate of polypharmacy (81%) in this hospitalised population was higher than previous studies in this setting (range 42–52%).^14 37^ 40% of patients globally are exposed to polypharmacy using the definition of five or more medicines as in this study. Estimates of the prevalence of polypharmacy vary from between 20% in the community setting and 52% in the inpatient setting using the broader WHO definition of multiple medicines at the same time or an excessive number of medicines.^14^ A previous study in Australian inpatients found that 42% of hospitalised older adults had polypharmacy, which is lower than our estimate.^37^ It is possible that the ageing population with higher rates of multimorbidity is leading to increased rates of polypharmacy in this population in part due to the use of guideline-directed medications for chronic diseases.

The prevalence of frailty (47%) in this study is higher than has been reported in the community setting (range 12–24%) but consistent with other studies of Australian inpatients (39–57%).^15 38 39^ 23% of similarly aged community-dwelling adults are reported to have frailty across multiple countries, although there is heterogeneity depending on the definition used.^15^ This is comparable to community-dwelling older adults in Australia, where 21% are reported to have frailty using the modified Fried Frailty Phenotype, which is based on self-assessment of weakness, exhaustion, slowness and low activity.^16^ Rates are higher in the Australian hospital setting with a previous study showing that 39% of hospitalised adults over 75 years had frailty using the hospital frailty risk score as in this study which is based on ICD-10 codes predominantly of functional impairment.^38^ The slightly lower rates of frailty in that study may have been due to the inclusion of a broader range of admitting unit than in this study. Other studies have shown that 57% of adult Australian inpatients had frailty using a multidomain frailty index from medical records (based on documentation, blood results and coding data).^39^ Both the hospital frailty risk score and the electronic frailty index have been developed to detect frailty using routinely collected data. However, these tools appear to identify different patients as being frail, although both are associated with prolonged length of stay.^38^ Given this, further studies are needed to confirm our estimate using other measures of frailty (such as the electronic frailty index) in addition to refining the tools being used to identify frail patients using routinely collected data.^40^

National guidelines for these cardiovascular comorbidities recommend treatment irrespective of the presence of frailty.^41^,^43^ However, it may be that clinicians are less likely to prescribe these drugs to adults with frailty due to concerns around medication-related harm, the generalisability of clinical trials to this population or time to benefit of these medicines.^11^ The clinical trials underlying these recommendations to use these medicines may not be generalisable to hospitalised adults who are older with higher rates of multimorbidity and frailty. For instance, it has been shown that one-third of patients with atrial fibrillation would not have met the criteria for the registrational trials for the direct oral anticoagulants.^33^ Alternatively, drug intolerance may have occurred, leading to cessation of these agents, as frailty might be a superior indicator of physiological vulnerability to medications compared with age alone.^44^ Conversely, patients with multimorbidity were more likely to receive multiple guideline-directed medications for cardiovascular conditions. Given the high prevalence of cardiovascular comorbidities, those with multimorbidity are more likely to have additional indications for more aggressive risk factor control to prevent cardiovascular events or anticoagulation to prevent stroke. This may explain why multimorbidity was associated with the use of antihypertensive, antithrombotic and lipid-lowering therapies.

There is some evidence to suggest that older adults, including those with frailty, may be at increased risk of medication-related harm or not receive the benefits of medication for these cardiovascular conditions. The use of antihypertensives in community-dwelling adults with frailty has been associated with an increased risk of harm, with observational data suggesting that a higher blood pressure may be protective.^45 46^ Similarly, in adults with frailty, dual antiplatelet therapy has been associated with an increased risk of adverse events and statins do not appear to confer mortality benefits in a systematic review of both prospective and retrospective studies.^47 48^ Studies have been conflicting on whether oral anticoagulants are associated with reduced risk of stroke or increased risk of bleeding in adults with frailty.^49 50^ However, one study has reported that the risk of bleeding or mortality is increased in those with cognitive impairment.^50^ Few of these studies have focused on the hospitalised population who have the highest rates of multimorbidity, polypharmacy and frailty. These patients are likely to be at high risk of medication-related harm due to the number of medications being prescribed, reduced physiological reserve and drug interactions.^51^,^53^ Prescribers should use this evidence in shared decision making with patients and their carers when considering the use of these medications for cardiovascular conditions in adults with frailty. These findings may influence guideline recommendations for these groups. Further outcome studies are needed to quantify the risks and benefits of cardiovascular medications in hospitalised adults.

This study has multiple strengths, including the large population of hospital inpatients reflecting real-world practices as well as the use of coding data to determine the presence of cardiovascular conditions. Given this dataset included multiple hospitals across Adelaide, these findings are likely to be generalisable to similar Australian hospitalised populations. However, there are limitations that should be acknowledged. First, medication orders at the time of hospital discharge may not equate to long-term medication use. This may have underestimated the rate of guideline-directed medication use, particularly if medications were temporarily put on hold during the admission and not restarted within the EMR prior to discharge. Second, two or more chronic medical conditions is a blunt definition of multimorbidity as it considers all conditions equally when these are likely to have different impact on prescribing practices. Despite this, it remains the most widely used and accepted definition of multimorbidity in the literature.^54^ Since this study was devised, more modern and comprehensive consensus definitions of multimorbidity including revised ICD-10 code lists have been published.^55 56^ Although these largely overlap with the chronic diseases included in the Barnett *et al*^27^ list, they include some additional conditions and may mean that the prevalence of multimorbidity based coding data was underestimated in this study. Third, the inability to include as needed medications in the count of medications may have underestimated the rates of polypharmacy. However, as needed medications are commonly excluded from polypharmacy counts using outpatient dispensing data because of the inability to determine the frequency of use. Fourth, the use of ICD-10 codes from hospital coding data may be insensitive for the detection of select chronic medical conditions, introducing misclassification bias. This was mitigated by confirming the prevalence of multimorbidity using RxRisk and only analysing prescribing patterns in those with ICD-10 codes for select cardiovascular diseases. Although this may have rendered the detection of those with a contraindication to anticoagulation insensitive, leading to an overestimate in the number of patients suitable for anticoagulation. Fifth, multimorbidity and frailty were both assessed using ICD-10 codes and the link between these is acknowledged. However, the ICD-10 codes had minimal overlap with the hospital frailty risk score focussing mainly on functional impairment and the multimorbidity count focussing on chronic medical conditions. Sixth, there may be unmeasured confounding in the logistic regressions to determine the factors associated with use of guideline-directed medicines. Factors such as socioeconomic status and ethnicity were not included in the analysis and this may have introduced confounding. Seventh, the use of patients who survived to discharge may have introduced collider bias as illness severity may influence both prescribing and survival. This would mean that associations may be found that would not have existed in the complete population. Finally, the generalisability of these findings to specialist hospital units and other settings including regional or private hospitals, community dwelling adults and international health systems is unclear.

In conclusion, there was a high prevalence of multimorbidity (69–94%), polypharmacy (81%) and frailty (45%) in the hospitalised population that exceeded previous estimates in the literature. More than 85% of patients with hypertension were taking an antihypertensive. More than 75% of patients with ischaemic heart disease were taking an antithrombotic or a lipid-lowering agent and more than 50% were taking an agent acting on the renin-angiotensin system. Over 70% of patients with atrial fibrillation without a contraindication to anticoagulation were taking an anticoagulant. Patients with multimorbidity were 11–51% more likely to be taking an antihypertensive, antithrombotic or lipid-lowering medicine for the respective cardiovascular conditions, whereas those with frailty were 31–48% less likely to be taking guideline-directed medicines for all conditions studied. Outcomes studies are needed to quantify the risks and benefits of these medicines in patients with multimorbidity or frailty who are not well represented in clinical trials. In the presence of specific comorbidities, the treatment of these cardiovascular conditions may be contraindicated or associated with an increased risk of harm. The findings of these studies will assist patients and prescribers in shared decision making when considering the use of these medications for cardiovascular conditions.

## Supplementary material

10.1136/bmjopen-2025-103243 online supplemental file 1

## Data Availability

Data are available upon reasonable request.
