# Supplementary material for: Prevalence of multimorbidity and uptake of guideline-directed medicines for cardiovascular conditions in Australian hospitalised adults: a cross-sectional study
Source: BMJ Open. 2026 Feb 10;16(2):e103243. doi: 10.1136/bmjopen-2025-103243 (PMC12911732; doi:10.1136/bmjopen-2025-103243)
Supplement: online supplemental file 1 [file bmjopen-16-2-s001.docx]

**Supplementary Table 1: Characteristics of Patients with Hypertension on an Antihypertensive**

|  |  | **On an Antihypertensive** | |  |
| --- | --- | --- | --- | --- |
| **Characteristic** | **Overall** N = 11,122*^1^* | **No**, N = 1641 (15%)*^1^* | **Yes**, N = 9481 (85%)*^1^* | **p-value***^2^* |
| Age, median (IQR) | 78 (68, 86) | 78 (68, 87) | 78 (68, 86) | 0.20 |
| Gender, n (%) |  |  |  | **<0.01** |
| Female | 5,346 (48%) | 734 (45%) | 4,612 (49%) |  |
| Male | 5,775 (52%) | 907 (55%) | 4,868 (51%) |  |
| Multimorbidity, n (%) |  |  |  | **0.03** |
| Present | 9,952 (89%) | 1,444 (88%) | 8,508 (90%) |  |
| Absent | 1,170 (11%) | 197 (12%) | 973 (10%) |  |
| Number of Long Term Medications, median (IQR) | 9 (6, 12) | 6 (4, 9) | 9 (7, 12) | **<0.01** |
| Frailty, n (%) | 5,120 (46%) | 873 (53%) | 4,247 (45%) | **<0.01** |
| Creatinine (umol/L) | 88 (68, 122) | 82 (64, 112) | 88 (69, 125) | **<0.01** |
| Hepatic Impairment, n (%) |  |  |  | 0.99 |
| Absent | 11,014 (99%) | 1,625 (99%) | 9,389 (99%) |  |
| Present | 108 (1.0%) | 16 (1.0%) | 92 (1.0%) |  |
| *^1^*Median (Q1, Q3); n (%) | | | | |
| *^2^*Wilcoxon rank sum test; Fisher's exact test; Pearson's Chi-squared test  Missing Values (Gender = 1, Creatinine = 22) | | | | |

**Supplementary Table 2: Characteristics of Patients with Ischaemic Heart Disease Based on whether on an Antiplatelet or Anticoagulant**

|  |  | **On Antiplatelet or Anticoagulant** | |  |
| --- | --- | --- | --- | --- |
| **Characteristic** | **Overall** N = 4,451*^1^* | **No**, N = 572 (13%)*^1^* | **Yes**, N = 3879 (87%)*^1^* | **p-value***^2^* |
| Age, median (IQR) | 78 (68, 86) | 81 (70, 88) | 77 (67, 85) | **<0.01** |
| Gender, n (%) |  |  |  | **<0.01** |
| Female | 1,530 (34%) | 241 (42%) | 1,289 (33%) |  |
| Male | 2,921 (66%) | 331 (58%) | 2,590 (67%) |  |
| Multimorbidity, n (%) |  |  |  | 0.59 |
| Present | 4,133 (93%) | 528 (92%) | 3,605 (93%) |  |
| Absent | 318 (7.1%) | 44 (7.7%) | 274 (7.1%) |  |
| Number of Long Term Medications, median (IQR) | 10 (7, 13) | 8 (5, 11) | 10 (8, 13) | **<0.01** |
| Frailty, n (%) | 1,764 (40%) | 263 (46%) | 1,501 (39%) | **<0.01** |
| Creatinine (umol/L) | 94 (73, 134) | 92 (69, 135) | 94 (74, 134) | 0.10 |
| Hepatic Impairment, n (%) |  |  |  | **0.03** |
| Absent | 4,418 (99%) | 563 (98%) | 3,855 (99%) |  |
| Present | 33 (0.7%) | 9 (1.6%) | 24 (0.6%) |  |
| *^1^*Median (Q1, Q3); n (%) | | | | |
| *^2^*Wilcoxon rank sum test; Pearson's Chi-squared test; Fisher's exact test  Missing Values (Creatinine = 2) | | | | |

**Supplementary Table 3: Characteristics of Patients with Ischaemic Heart Disease based on whether on Any Lipid Lowering Agent**

|  |  | **On Lipid Lowering Agent** | |  |
| --- | --- | --- | --- | --- |
| **Characteristic** | **Overall** N = 4,451*^1^* | **No**, N = 985 (22%)*^1^* | **Yes**, N = 3466 (78%)*^1^* | **p-value***^2^* |
| Age, median (IQR) | 78 (68, 86) | 83 (73, 90) | 76 (66, 84) | **<0.01** |
| Gender, n (%) |  |  |  | **<0.01** |
| Female | 1,530 (34%) | 425 (43%) | 1,105 (32%) |  |
| Male | 2,921 (66%) | 560 (57%) | 2,361 (68%) |  |
| Multimorbidity, n (%) |  |  |  | **<0.01** |
| Present | 4,133 (93%) | 936 (95%) | 3,197 (92%) |  |
| Absent | 318 (7.1%) | 49 (5.0%) | 269 (7.8%) |  |
| Number of Long Term Medications, median (IQR) | 10 (7, 13) | 9 (6, 12) | 10 (8, 13) | **<0.01** |
| Frailty, n (%) | 1,764 (40%) | 527 (54%) | 1,237 (36%) | **<0.01** |
| Creatinine (umol/L) | 94 (73, 134) | 94 (71, 137) | 94 (74, 134) | 0.48 |
| Hepatic Impairment, n (%) |  |  |  | 0.77 |
| Absent | 4,418 (99%) | 977 (99%) | 3,441 (99%) |  |
| Present | 33 (0.7%) | 8 (0.8%) | 25 (0.7%) |  |
| *^1^*Median (Q1, Q3); n (%) | | | | |
| *^2^*Wilcoxon rank sum test; Pearson's Chi-squared test; Fisher's exact test  Missing Values (Creatinine = 2) | | | | |

**Supplementary Table 4: Characteristics of Patients with Ischaemic Heart Disease based on whether on any agent acting on the Renin-Angiotensin System**

|  |  | **On Agent Acting on the Renin-Angiotensin System** | |  |
| --- | --- | --- | --- | --- |
| **Characteristic** | **Overall** N = 4,451*^1^* | **No**, N = 1870 (42%)*^1^* | **Yes**, N = 2581 (58%)*^1^* | **p-value***^2^* |
| Age, median (IQR) | 78 (68, 86) | 80 (70, 87) | 76 (66, 85) | **<0.01** |
| Gender, n (%) |  |  |  | **<0.01** |
| Female | 1,530 (34%) | 694 (37%) | 836 (32%) |  |
| Male | 2,921 (66%) | 1,176 (63%) | 1,745 (68%) |  |
| Multimorbidity, n (%) |  |  |  | 0.85 |
| Present | 4,133 (93%) | 1,738 (93%) | 2,395 (93%) |  |
| Absent | 318 (7.1%) | 132 (7.1%) | 186 (7.2%) |  |
| Number of Long Term Medications, median (IQR) | 10 (7, 13) | 10 (7, 13) | 10 (8, 13) | **<0.01** |
| Frailty, n (%) | 1,764 (40%) | 905 (48%) | 859 (33%) | **<0.01** |
| Creatinine (umol/L) | 94 (73, 134) | 95 (72, 145) | 93 (74, 128) | **0.02** |
| Hepatic Impairment, n (%) |  |  |  | 0.45 |
| Absent | 4,418 (99%) | 1,854 (99%) | 2,564 (99%) |  |
| Present | 33 (0.7%) | 16 (0.9%) | 17 (0.7%) |  |
| *^1^*Median (Q1, Q3); n (%) | | | | |
| *^2^*Wilcoxon rank sum test; Pearson's Chi-squared test  Missing Values (Creatinine = 2) | | | | |

**Supplementary Table 5: Characteristics of Patients with Atrial Fibrillation without Contraindication to Anticoagulation based on Anticoagulation Status**

|  |  | **Anticoagulated** | |  |
| --- | --- | --- | --- | --- |
| **Characteristic** | **Overall** N = 1,770*^1^* | **No**, N = 493 (28%)*^1^* | **Yes**, N = 1277 (72%)*^1^* | **p-value***^2^* |
| Age, median (IQR) | 81 (71, 87) | 80 (70, 88) | 81 (71, 87) | 0.89 |
| Gender, n (%) |  |  |  | 0.51 |
| Female | 848 (48%) | 230 (47%) | 618 (48%) |  |
| Male | 922 (52%) | 263 (53%) | 659 (52%) |  |
| Multimorbidity, n (%) |  |  |  | **0.02** |
| Present | 1,659 (94%) | 451 (91%) | 1,208 (95%) |  |
| Absent | 111 (6.3%) | 42 (8.5%) | 69 (5.4%) |  |
| Number of Long Term Medications, median (IQR) | 9 (7, 12) | 8 (6, 11) | 9 (7, 12) | **<0.01** |
| Frailty, n (%) | 1,012 (57%) | 313 (63%) | 699 (55%) | **<0.01** |
| Creatinine (umol/L) | 91 (69, 125) | 89 (66, 134) | 92 (72, 122) | 0.50 |
| Hepatic Impairment, n (%) |  |  |  | 0.15 |
| Absent | 1,760 (99%) | 488 (99%) | 1,272 (100%) |  |
| Present | 10 (0.6%) | 5 (1.0%) | 5 (0.4%) |  |
| *^1^*Median (Q1, Q3); n (%) | | | | |
| *^2^*Wilcoxon rank sum test; Pearson's Chi-squared test; Fisher's exact test  Missing Values (Creatinine = 1) | | | | |

**Supplementary Table 6: Logistic regression of the factors associated with use of an antihypertensive in those with hypertension**

| **Characteristic** | **OR***^1^* | **95% CI***^1^* | **p-value** |
| --- | --- | --- | --- |
| Age (years) | 1.00 | 0.99, 1.00 | 0.80 |
| Male Gender | 0.81 | 0.72, 0.91 | **<0.01** |
| Multimorbidity | 1.51 | 1.27, 1.80 | **<0.01** |
| Number of Long Term Medications | 1.25 | 1.23, 1.27 | **<0.01** |
| Frailty | 0.57 | 0.50, 0.64 | **<0.01** |
| Log of Serum Creatinine | 1.30 | 1.17, 1.46 | **<0.01** |
| Hepatic Impairment | 0.83 | 0.49, 1.50 | 0.51 |
| *^1^*OR = Odds Ratio, CI = Confidence Interval | | | |

**Supplementary Table 7: Logistic regression of the factors associated with use of antiplatelet or anticoagulant in those with ischaemic heart disease**

| **Characteristic** | **OR***^1^* | **95% CI***^1^* | **p-value** |
| --- | --- | --- | --- |
| Age (years) | 0.99 | 0.98, 1.00 | **0.02** |
| Male Gender | 1.61 | 1.33, 1.95 | **<0.01** |
| Multimorbidity | 1.49 | 1.05, 2.14 | **0.03** |
| Number of Long Term Medications | 1.24 | 1.20, 1.28 | **<0.01** |
| Frailty | 0.69 | 0.57, 0.84 | **<0.01** |
| Log of Serum Creatinine | 0.90 | 0.76, 1.08 | 0.26 |
| Hepatic Impairment | 0.28 | 0.13, 0.66 | **<0.01** |
| *^1^*OR = Odds Ratio, CI = Confidence Interval | | | |

**Supplementary Table 8: Logistic regression of the factors associated with the use of lipid lowering therapy in those with ischaemic heart disease**

| **Characteristic** | **OR***^1^* | **95% CI***^1^* | **p-value** |
| --- | --- | --- | --- |
| Age (years) | 0.97 | 0.96, 0.97 | **<0.01** |
| Male Gender | 1.56 | 1.34, 1.83 | **<0.01** |
| Multimorbidity | 1.89 | 1.36, 2.67 | **<0.01** |
| Number of Long Term Medications | 1.17 | 1.14, 1.19 | **<0.01** |
| Frailty | 0.52 | 0.44, 0.61 | **<0.01** |
| Log of Serum Creatinine | 0.95 | 0.82, 1.10 | 0.48 |
| Hepatic Impairment | 0.65 | 0.29, 1.60 | 0.32 |
| *^1^*OR = Odds Ratio, CI = Confidence Interval | | | |

**Supplementary Table 9: Logistic regression of the factors associated with use of an agent acting on the renin-angiotensin system in those with ischaemic heart disease**

| **Characteristic** | **OR***^1^* | **95% CI***^1^* | **p-value** |
| --- | --- | --- | --- |
| Age (years) | 0.99 | 0.98, 0.99 | **<0.01** |
| Male Gender | 1.23 | 1.08, 1.40 | **<0.01** |
| Multimorbidity | 0.93 | 0.73, 1.19 | 0.56 |
| Number of Long Term Medications | 1.06 | 1.04, 1.08 | **<0.01** |
| Frailty | 0.56 | 0.50, 0.64 | **<0.01** |
| Log of Serum Creatinine | 0.78 | 0.70, 0.87 | **<0.01** |
| Hepatic Impairment | 0.72 | 0.35, 1.46 | 0.35 |
| *^1^*OR = Odds Ratio, CI = Confidence Interval | | | |

**Supplementary Table 10: Logistic regression of the factors associated with anticoagulation in those with atrial fibrillation without a contraindication to anticoagulation**

| **Characteristic** | **OR***^1^* | **95% CI***^1^* | **p-value** |
| --- | --- | --- | --- |
| Age (years) | 1.01 | 1.00, 1.02 | 0.23 |
| Male Gender | 1.02 | 0.81, 1.27 | 0.88 |
| Multimorbidity | 1.44 | 0.95, 2.17 | 0.08 |
| Number of Long Term Medications | 1.09 | 1.06, 1.12 | **<0.01** |
| Frailty | 0.66 | 0.53, 0.83 | **<0.01** |
| Log of Serum Creatinine | 0.74 | 0.61, 0.90 | **<0.01** |
| Hepatic Impairment | 0.38 | 0.10, 1.42 | 0.14 |
| *^1^*OR = Odds Ratio, CI = Confidence Interval | | | |

**Appendix A: Admitting Units Included**

| **Unit Category** | **Admitting Unit** |
| --- | --- |
| Medicine | QEH Acute Medical Unit / 001 |
|  | QEH Cardiac - Medical / 012 |
|  | QEH Endocrine / 015 |
|  | QEH Gastroenterology / 017 |
|  | QEH General Medical / 006 |
|  | QEH General Medical / 007 |
|  | QEH General Medical / 008 |
|  | QEH General Medical / 009 |
|  | QEH Geriatric Medicine / 071 |
|  | QEH Infectious Diseases / 019 |
|  | QEH Respiratory / 025 |
|  | QEH Rheumatology / 026 |
|  | RAH ACS Cardiology / 016 |
|  | RAH Arrhythmia Cardiology / 012 |
|  | RAH Dermatology / 014 |
|  | RAH EDGE / 173 |
|  | RAH Endocrine / 018 |
|  | RAH Gastroenterology / 004 |
|  | RAH General Medical / 101 |
|  | RAH General Medical / 102 |
|  | RAH General Medical / 103 |
|  | RAH General Medical / 104 |
|  | RAH General Medical / 105 |
|  | RAH General Medical / 106 |
|  | RAH General Medical / 107 |
|  | RAH Geriatric / 070 |
|  | RAH Infectious Diseases / 022 |
|  | RAH Neurology / 027 |
|  | RAH Neurology B / 093 |
|  | RAH Renal Inpatient / 026 |
|  | RAH Rheumatology / 017 |
|  | RAH Stroke / 087 |
|  | RAH Structural Cardiology / 013 |
|  | RAH Thoracic / 060 |
|  | RAH ED CDU / 008 |
| Mental Health | QEH Acute MH / 077 |
|  | QEH MH Cramond Acute / 078 |
|  | RAH Acute MH / 092 |
|  | RAH Short Stay MH / 278 |
| Surgery | QEH Breast Endocrine / 037 |
|  | QEH Colorectal / 036 |
|  | QEH ENT / 044 |
|  | RAH General Surgery / 032 |
|  | RAH General Surgery / 033 |
|  | RAH General Surgery / 036 |
|  | RAH General Surgery / 037 |
|  | QEH Gynaecology / 067 |
|  | QEH Ortho Geriatrics / 053 |
|  | QEH Orthopaedic Surgery / 051 |
|  | QEH Orthopaedic Surgery / 052 |
|  | QEH Plastic Surgery / 054 |
|  | QEH Upper GI / 035 |
|  | QEH Urology / 056 |
|  | RAH Breast Endocrine / 035 |
|  | RAH Cardiac - Surgical / 059 |
|  | RAH Craniofacial / 056 |
|  | RAH ENT / 054 |
|  | RAH Gynaecology / 080 |
|  | RAH Ophthalmology / 043 |
|  | RAH Ophthalmology / 047 |
|  | RAH Ophthalmology / 048 |
|  | RAH Oral Surgery / 057 |
|  | RAH Orthopaedic Surgery / 051 |
|  | RAH Orthopaedic Surgery / 052 |
|  | RAH Plastic Surgery / 058 |
|  | RAH Urology / 061 |
|  | RAH Vascular / 040 |

**Appendix B: Cardiovascular Comorbidities of Interest**

| **Cardiovascular Conditions of Interest** | **ICD-10 Codes** |
| --- | --- |
| Hypertension | I10, I11.0, I11.9, I12.0, I12.9, I13.0, I13.1, I13.2, I13.9, I15.0, I15.1, I15.2, I15.8, I15.9 |
| Ischaemic heart disease | I20.0, I20.1, I20.8, I20.9, I21.0, I21.1, I21.2, I21.3, I21.4, I21.9, I22.0, I22.1, I22.8, I22.9, I23.0, I23.1, I23.2, I23.3, I23.4, I23.5, I23.6, I23.8, I24.0, I24.1, I24.8, I24.9, I25.0, I25.10, I25.11, I25.12, I25.13, I25.2, I25.3, I25.4, I25.5, I25.6, I25.8, I25.9 |
| Atrial fibrillation without a contraindication to anticoagulation | I48.0, I48.1, I48.2, I48.3, I48.4, I48.9  Excluding blood dyscrasias, cirrhosis, hepatic decompensation, intracranial haemorrhage, intracranial mass and prior gastrointestinal haemorrhage (see Appendix D) |

**Appendix C: Identification of Hepatic Impairment**

| **Group of Conditions** | **ICD-10 Codes** |
| --- | --- |
| Hepatic impairment | K70.2, K70.3, K71.7, K72.1, K72.9, K73.0, K73.1, K73.2, K73.8, K73.9, K74.0, K74.2, K74.3, K74.4, K74.5, K74.6, K76.6, K76.7 |
| Based on Sundararajan et al. 2004 | |

**Appendix D: ATC Codes for Guideline-Directed Medications**

| **Cardiovascular Condition** | **Guideline-Directed Medication** | **ATC Codes** |
| --- | --- | --- |
| Hypertension | Antihypertensives |  |
|  | Angiotensin receptor blocker | C09C, C09D |
|  | Dihydropyridine calcium channel blockers | C08CA |
|  | Angiotensin converting enzyme inhibitor | C09A, C09B |
|  | Thiazide or thiazide-like diuretics | C03A, C03BA |
| Ischaemic heart disease | Antiplatelet or anticoagulant |  |
|  | Aspirin | B01AC06, B01AC56 |
|  | Clopidogrel | B01AC04 |
|  | Ticagrelor | B01AC24 |
|  | Rivaroxaban | B01AF01 |
|  | Lipid lowering agents |  |
|  | Statins | C10AA, C10BX |
|  | Ezetimibe | C10AX09 |
|  | PCSK9 Inhibitors | C10AX13, C10AX14 |
|  | Agents acting on the renin-angiotensin system |  |
|  | Angiotensin converting enzyme inhibitor | C09A, C09B |
|  | Angiotensin receptor blocker | C09C, C09D |
| Atrial fibrillation | Anticoagulants |  |
|  | Apixaban | B01AF02 |
|  | Rivaroxaban | B01AF01 |
|  | Warfarin | B01AA |
|  | Therapeutic enoxaparin | B01AB05 |
|  | Dabigatran | B01AE07 |

**Appendix E: Contraindications to Anticoagulation**

| **Group of Conditions** | **ICD-10 Codes** |
| --- | --- |
| Blood dyscrasias | C81.0, C81.1, C81.2, C81.3, C81.4, C81.7, C81.9, C82.0, C82.1, C82.2, C82.3, C82.4, C82.5, C82.6, C82.7, C82.9, C83.0, C83.1, C83.3, C83.5, C83.7, C83.8, C83.9, C84.0, C84.1, C84.4, C84.5, C84.6, C84.7, C84.8, C84.9, C85.1, C85.2, C85.7, C85.9, C86.0, C86.1, C86.2, C86.3, C86.4, C86.5, C86.6, C88.00, C88.01, C88.20, C88.21, C88.30, C88.31, C88.40, C88.41, C88.70, C88.71, C88.90, C88.91, C90.00, C90.01, C90.10, C90.11, C90.20, C90.21, C90.30, C90.31, C91.00, C91.01, C91.10, C91.11, C91.30, C91.31, C91.40, C91.41, C91.50, C91.51, C91.60, C91.61, C91.70, C91.71, C91.80, C91.81, C91.90, C91.91, C92.00, C92.01, C92.10, C92.11, C92.20, C92.21, C92.30, C92.31, C92.40, C92.41, C92.50, C92.51, C92.60, C92.61, C92.70, C92.71, C92.80, C92.81, C92.90, C92.91, C93.00, C93.01, C93.10, C93.11, C93.30, C93.31, C93.70, C93.71, C93.90, C93.91, C94.00, C94.01, C94.20, C94.21, C94.30, C94.31, C94.40, C94.41, C94.60, C94.61, C94.70, C94.71, C95.00, C95.01, C95.10, C95.11, C95.70, C95.71, C95.90, C95.91, C96.0, C96.2, C96.4, C96.5, C96.6, C96.7, C96.8, C96.9, D50.0, D50.1, D50.8, D50.9, D51.0, D51.1, D51.2, D51.3, D51.8, D51.9, D52.0, D52.1, D52.8, D52.9, D53.0, D53.1, D53.2, D53.8, D53.9, D55.0, D55.1, D55.2, D55.3, D55.8, D55.9, D56.0, D56.1, D56.2, D56.3, D56.4, D56.8, D56.9, D57.0, D57.1, D57.2, D57.3, D57.8, D58.0, D58.1, D58.2, D58.8, D58.9, D59.0, D59.1, D59.2, D59.3, D59.4, D59.5, D59.6, D59.8, D59.9, D69.3, D69.4, D69.5, D69.6 |
| Cirrhosis | K70.3, K74.0, K74.3, K74.4, K74.5, K74.6 |
| Hepatic decompensation | I85.0, I85.9, K65.9, K65.11, K65.3, K65.8, K65.21, K65.22, K65.23, K65.24, K65.9, K65.19, K65.29, K67.0, K67.1, K67.2, K67.3, K67.8, K76.7, R18 |
| Intracranial haemorrhage | I60.0, I60.1, I60.2, I60.3, I60.4, I60.5, I60.6, I60.7, I60.8, I60.9, I61.0, I61.1, I61.2, I61.3, I61.4, I61.5, I61.6, I61.8, I61.9, I62.0, I62.1, I62.9, S06.23, S06.33, S06.34, S06.4, S06.5, S06.6, S06.4 |
| Intracranial mass | C71.0, C71.1, C71.2, C71.3, C71.4, C71.5, C71.6, C71.7, C71.8, C71.9, C79.3, D33.0, D33.1, D33.2 |
| Prior gastrointestinal haemorrhage | K25.0, K25.2, K25.4, K25.6, K26.0, K26.2, K26.4, K26.6, K27.0, K27.2, K27.4, K27.6, K28.0, K28.2, K28.4, K28.6, K29.0, K29.21, K29.31, K29.41, K29.51, K29.61, K29.71, K29.81, K29.91, K31.81, K31.82, K55.21, K55.22, K55.31, K55.32, K62.5, K92.0, K92.1, K92.2 |
| Adapted to ICD-10 from Steinberg et al. 2019 | |
