# Supplementary material for: Prevalence of multimorbidity and uptake of guideline-directed medicines for cardiovascular conditions in Australian hospitalised adults: a cross-sectional study
Source: BMJ Open. 2026 Feb 10;16(2):e103243. doi: 10.1136/bmjopen-2025-103243 (PMC12911732; doi:10.1136/bmjopen-2025-103243)
Supplement: Supplementary file 2 [file bmjopen-2025-103243.reviewer_comments.pdf]

PEER REVIEW HISTORY

BMJ Open publishes all reviews undertaken for accepted manuscripts. Reviewers are asked to complete a checklist review form and are provided with free text boxes to elaborate on their assessment. These free text comments are reproduced below.

ARTICLE DETAILS

Title (Provisional)

Prevalence of multimorbidity and uptake of guideline-directed medicines for cardiovascular conditions in Australian hospitalised adults: a cross-sectional study

Authors

Inglis, Joshua; Caughey, Gillian Elizabeth; Liew, Danny; Shakib, Sepehr

---

VERSION 1 - REVIEW

**Reviewer**	**1**
**Name**	**Prazeres, Filipe**
**Affiliation**	**Universidade da Beira Interior Faculdade de Ciencias da Saude**
**Date**	**25-Jul-2025**
**COI**	**None**

Thank you for the opportunity to review this manuscript. The paper is clearly written, methodologically sound, and the key findings are presented in a coherent way. All major sections of the manuscript appear appropriate and well-structured.

However, I would encourage the authors to clarify how frailty was defined and measured in the study. Given the importance of frailty as a clinical concept, distinct from, but often overlapping with, multimorbidity, it is essential to specify the criteria, tools, or data sources used to identify frail individuals. This will strengthen the transparency and reproducibility of your analysis.

In addition, the contrasting findings between multimorbidity and frailty in relation to prescribing patterns are striking and merit further reflection. In multimorbidity, the key challenge is often managing multiple appropriate treatments while avoiding overburdening the patient, particularly with respect to polypharmacy. Frailty, by contrast, introduces a different clinical dilemma: whether the risks of treatment outweigh the potential benefits, often leading to intentional under-treatment. It would be helpful for the authors to expand on this distinction in the discussion and consider how it might inform future clinical decision-making or guideline development for these two complex patient populations.

Thank you again for your contribution to this area of research.

Sincerely.

**Reviewer**	**2**
**Name**	**Hall, Marlous**
**Affiliation**	**University of Leeds**
**Date**	**24-Sep-2025**
**COI**	**None**

This is a reasonably sized study of almost 24,000 individuals with data from two hospitals in Adelaide to understand multimorbidity and polypharmacy in relation to cardiovascular disease. However I have concerns over the reporting standards not being met (detailed below) and about the overall definition of multimorbidity which uses a set of conditions from a now very dated paper of 2012. There has been a huge body of literature and many more modern approaches to define multimorbidity and importantly the conditions which contribute to multimorbidity which the authors should consider carefully (see for example a full review of multimorbidity in cardiovascular disease here which highlights the importance of using suitable & comprehensive MM definitions: https://www.jacc.org/doi/10.1016/j.jacadv.2025.102006). Furthermore - the clinical utility of the basic definition of 2+LTCs is not clear. We know that someone with hypertension plus diabetes is very different to someone with COPD and heart failure - but are both classed as "multimorbid". More sophisticated definitions are needed to address multimorbidity in particular in a study such as this which is seeking to understand the impact of multimorbidity on treatments - and categorising all forms of multimorbidity into one group is much less informative.

The authors have uploaded a STROBE statement but there are many elements completed with a "N/A" which means that not all of the required reporting standards have been met. These are fundamental aspects of the reporting of observational research. Those listed as N/A that require attention and addressing are listed below. All research of this nature is prone to a wide range of biases and efforts made to reduce these biases (e.g. confounder adjustment, dealing with missing data, selection bias and more) should be discussed directly in the manuscript. Missing data needs to be discussed in the manuscript - even if to say there were no missing data (which is rarely the case). If there are data missing - then this needs to be addressed suitably to minimise any potential biases that may arise. Flow diagrams are incredibly helpful to understand the original data extract and any exclusions that were made to arrive at the final analytical cohort (e.g. you have 24,000 individuals from the bigger set of 95K + 40K individuals quoted annually at each hospital). This level of detail will help ensure reproducibility of the work. Sensitivity analyses should be mentioned even if the authors decided not to conduct any. Were there no decision points in the analyses process that would benefit from sensitivity analyses? Finally - discussing the generalisability of findings is critical and should not be overlooked.

Bias #9 Describe any efforts to address potential sources of bias N/A

Statistical methods #12c Explain how missing data were addressed N/A

Statistical methods #12d If applicable, describe analytical methods taking account of sampling strategy N/A

Statistical methods #12e Describe any sensitivity analyses N/A

Participants #13c. Consider use of a flow diagram N/A

Descriptive data #14b. Indicate number of participants with missing data for each variable of interest

Generalisability #21. Discuss the generalisability (external validity) of the study results

I have not conducted a further review of the work at this stage given there are fundamental missing aspects in terms of reporting and the concerns over the definition of multimorbidity. I will be happy to review the methodological approaches and the paper in further detail if the above concerns are addressed.

VERSION 1 - AUTHOR RESPONSE

Reviewer: 1
Prof. Filipe Prazeres, Universidade da Beira Interior Faculdade de Ciencias da Saude, CINTESIS
Comments to the Author:

Thank you for the opportunity to review this manuscript. The paper is clearly written, methodologically sound, and the key findings are presented in a coherent way. All major sections of the manuscript appear appropriate and well-structured.

Thank you.

However, I would encourage the authors to clarify how frailty was defined and measured in the study. Given the importance of frailty as a clinical concept, distinct from, but often overlapping with, multimorbidity, it is essential to specify the criteria, tools, or data sources used to identify frail individuals. This will strengthen the transparency and reproducibility of your analysis.

Frailty was defined using the hospital frailty risk score. This is outlined in the Methods as follows:

“The hospital frailty risk score, which uses ICD-10 codes for markers of frailty (e.g. cognitive impairment, functional dependence, falls and fractures) was used to identify those with frailty(28). Individuals with an intermediate or high risk of frailty using this score were considered to be frail consistent with the derivation study.” (p. 9, line 165-172)

This definition of frailty has been validated (see reference 28) and is widely used in health outcomes research. It uses ICD-10 codes making it reproducible in any health setting using these codes.

In addition, the contrasting findings between multimorbidity and frailty in relation to prescribing patterns are striking and merit further reflection. In multimorbidity, the key challenge is often managing multiple appropriate treatments while avoiding overburdening the patient, particularly with respect to polypharmacy. Frailty, by contrast, introduces a different clinical dilemma: whether the risks of treatment

outweigh the potential benefits, often leading to intentional under-treatment. It would be helpful for the authors to expand on this distinction in the discussion and consider how it might inform future clinical decision-making or guideline development for these two complex patient populations.

These findings and the implications have been extensively discussed in paragraphs 4 and 5 of the Discussion.

An additional section has been added on the associations with multimorbidity in paragraph 4 of the Discussion. This reads:

“Conversely, patients with multimorbidity were more likely to receive multiple guideline-directed medications for cardiovascular conditions. Given the high prevalence of cardiovascular comorbidities, those with multimorbidity are more likely to have additional indications for more aggressive risk factor control to prevent cardiovascular events or anticoagulation to prevent stroke. This may explain why multimorbidity was associated with the use of antihypertensive, antithrombotic and lipid lowering therapies.” (p. 19, line 376-382)

An additional sentence on the implications for prescribers has been added to paragraph 5.

“Prescribers should use this evidence in shared decision making with patients and their carers when considering the use of these medications for cardiovascular conditions in adults with frailty. These findings may influence guideline recommendations for these groups.” (p. 20, line 398-401)

Reviewer: 2
Dr. Marlous Hall, University of Leeds
Comments to the Author:
This is a reasonably sized study of almost 24,000 individuals with data from two hospitals in Adelaide to understand multimorbidity and polypharmacy in relation to cardiovascular disease. However I have concerns over the reporting standards not being met (detailed below) and about the overall definition of multimorbidity which uses a set of conditions from a now very dated paper of 2012. There has been a huge body of literature and many more modern approaches to define multimorbidity and importantly the conditions which contribute to multimorbidity which the authors should consider carefully (see for example a full review of multimorbidity in cardiovascular disease here which highlights the importance of using suitable & comprehensive MM definitions: https://www.jacc.org/doi/10.1016/j.jacadv.2025.102006). Furthermore - the clinical utility of the basic definition of 2+LTCs is not clear. We know that someone with hypertension plus diabetes is very different to someone with COPD and heart failure - but are both classed as "multimorbid". More sophisticated definitions are needed to address multimorbidity in particular in a study such as this which is seeking to understand the impact of multimorbidity on treatments - and categorising all forms of multimorbidity into one group is much less informative.

The World Health Organisation definition of multimorbidity is the “coexistence of two or more chronic conditions in the same individual”.

One of the authors has recently conducted a systematic review on definitions of multimorbidity (unpublished data). This found that two or more chronic

medical conditions is the most commonly used and widely accepted definition in the literature.

While we acknowledge that there are other approaches and this is a blunt definition, it is widely accepted and used in the literature. We do not believe using a different definition would have changed the conclusions.

Nevertheless, we have added the definition as a limitation:

"Secondly, two or more chronic medical conditions is a blunt definition of multimorbidity as it considers all conditions equally when these likely have different impact on prescribing practices. Despite this, it remains the most widely used and accepted definition of multimorbidity in the literature.(54)" (p. 20, line 411-414)

The authors have uploaded a STROBE statement but there are many elements completed with a "N/A" which means that not all of the required reporting standards have been met. These are fundamental aspects of the reporting of observational research. Those listed as N/A that require attention and addressing are listed below. All research of this nature is prone to a wide range of biases and efforts made to reduce these biases (e.g. confounder adjustment, dealing with missing data, selection bias and more) should be discussed directly in the manuscript. Missing data needs to be discussed in the manuscript - even if to say there were no missing data (which is rarely the case). If there are data missing - then this needs to be addressed suitably to minimise any potential biases that may arise. Flow diagrams are incredibly helpful to understand the original data extract and any exclusions that were made to arrive at the final analytical cohort (e.g. you have 24,000 individuals from the bigger set of 95K + 40K individuals quoted annually at each hospital). This level of detail will help ensure reproducibility of the work. Sensitivity analyses should be mentioned even if the authors decided not to conduct any. Were there no decision points in the analyses process that would benefit from sensitivity analyses? Finally - discussing the generalisability of findings is critical and should not be overlooked.

Bias #9 Describe any efforts to address potential sources of bias N/A

This has been added to the Methods:

"The inclusion criteria were all adults aged ≥45 years with first admissions via the emergency department under specific units who manage patients with general comorbidities (see Appendix A), such as General Medicine, General Surgery and Geriatrics. This was to avoid the overrepresentation of frequently hospitalised individuals." (p. 20, line 136-142)

Statistical methods #12c Explain how missing data were addressed N/A

This has been added to the Methods:

"Missing data were minimal (<5%) due to the structured nature of the electronic medical record. These were limited to gender and serum creatinine. Individuals with missing values were excluded from the regression analyses. Given the small proportion of missing data, imputation was not performed. This is not expected to have influenced the outcomes." (p. 11, line 207-211)

Statistical methods #12d If applicable, describe analytical methods taking account of sampling strategy N/A

This has been added to the Methods:

"Given all first hospitalisations during the study period were included, no further sampling strategy was applied." (p. 8, line 133-134)

Statistical methods #12e Describe any sensitivity analyses N/A

This sentence has been added to the Methods:

"No sensitivity analyses were performed, as results were expected to be robust given the low proportion of missing data and inclusion of all eligible hospitalisations." (p. 11-12, line 213-215)

Participants #13c. Consider use of a flow diagram N/A

A flow diagram has not been included as all eligible hospitalisations were included, and exclusions were limited to a small number (<5%) with missing data. The derivation of the cohort is explained in the Methods.

Descriptive data #14b. Indicate number of participants with missing data for each variable of interest

This has been indicated in Table 1 (1 individual with missing Gender) and Supplementary Tables 1-3 (total of 22 individuals with missing creatinine values).

Generalisability #21. Discuss the generalisability (external validity) of the study results

This has been added to the Discussion as follows:

"Given this dataset included multiple hospitals across Adelaide, these findings are likely to be generalisable to similar Australian hospital populations." (p. 20, line 406-407)

"Finally, the generalisability of these findings to other settings including regional or private hospitals, community dwelling adults and international health systems is unclear." (p. 21, line 431-432)

Thank you for your consideration of this revised manuscript.

VERSION 2 - REVIEW

**Reviewer**	**1**
**Name**	**Prazeres, Filipe**

Affiliation **Universidade da Beira Interior Faculdade de Ciencias da Saude**

Date **24-Oct-2025**

COI

Thank you for replying to my questions, nothing else to state.

Reviewer **2**

Name **Hall, Marlous**

Affiliation **University of Leeds**

Date **12-Nov-2025**

COI

Whilst some elements raised in my review have been addressed or rebutted suitably - there are a few aspects which are still outstanding and I feel these don't quite meet the expected reporting standards. I have also provided some further clarity on my point about the definition of multimorbidity which may have been misinterpreted by the authors in my earlier review - and some further thought on the implications of this in the manuscript would be beneficial.

1. Regarding the STROBE/RECORD statement "Bias #9 Describe any efforts to address potential sources of bias". The authors have added "This was to avoid the overrepresentation of frequently hospitalised individuals." (p. 20, line 136-142) alongside their inclusion criteria in the methods - however I do not feel this suitably describes efforts to address potential sources of bias.

As per my original comment - observational research using EHRs are prone to a wide range of biases. It is important, and expected as outlined in the reporting guidelines, to ensure there are efforts made to reduce these biases and that such efforts are reported directly and clearly in the manuscript (usually in methods and discussion). The authors have not expressly listed their thought process on confounder adjustment (or mentioned confounders at all), nor have they mentioned efforts to reduce other potential sources of bias such as selection bias.

Specifically - the statistical analyses section states that "Multivariate logistic regression was used to determine the association between multimorbidity and frailty on the use of guideline-directed medicines when controlling for the covariates of age, gender, number of regular medicines, the logarithm of serum creatinine on admission and hepatic impairment." The use of the term covariates is a little ambiguous - and it would be better if the authors could be more explicit about whether they consider all the covariates included to be confounders of the exposure/outcome relationship? A brief explanation providing justification for the selection of this particular set of confounders in the methods is also needed, and in the discussion it would be appropriate to address whether there were other potential confounders which may influence the exposure/outcome relationship that were

unable to be included (e.g. due to poor data quality or lack of availability of information). For example ethnicity, socioeconomic deprivation, changes in clinical practice or recording over time are a few potential confounders that haven't been mentioned or included. It is important to highlight potential for residual confounding in the findings and highlight that their omission may have resulted in some bias as a study limitation. Incorporating these elements would better address the reporting standards item on bias - which has been designed to improve the scientific integrity of published observational research.

2. Very minor comment on the same sentence - should this instead read "the association between multimorbidity and frailty *with* the use of guideline-directed medicines..."?

3. Multimorbidity definition. I wanted to clarify with the authors that I was not disputing the WHO's definition of multimorbidity being the co-existence of two or more long term conditions and apologise if the way I phrased my review caused confusion. Rather I want to highlight that the way that this definition of 2+LTCs is applied or implemented in research can cause problems. My concern is two-fold 1) No two LTCs are the same yet they all are aggregated in the same way with the same weight - there is a huge loss of valuable information here by reducing this to a binary concept which does not account for the specific conditions individuals have. However - I note the authors have now added a statement to acknowledge this limitation of their approach to address this point. 2) Another concern is the heterogeneity in the number of LTCs included in the count of 2+LTCs in research and alongside that a lack of transparency of how and why the choice of LTCs has been arrived at - this element has not yet been addressed by the authors. The paper I cited in my initial review highlights very clearly that the prevalence and impact of 2+LTCs is heavily influenced by the specific conditions included (e.g. one paper might include a limited set of conditions, another might comprehensively include LTCs relating to all body systems). In this study - the authors have cited the Barrett et al paper from 2012 to inform their choice of LTCs to include. This was a highly influential paper - laying the foundations for much subsequent multimorbidity research. However it would be good for the authors to justify their choice of these conditions more strongly - given the field of research has significantly advanced in recent years and there have been many subsequent papers reviewing the definitions of multimorbidity through delphi processes aiming to reach a consensus (e.g. one example, but not the only one, is the study by Ho et al). I very much appreciate that there remain multiple ways to define multimorbidity despite the efforts of previous consensus studies - but this is all the more reason to provide a discussion and clear justification for the choice of included LTCs included in this study. Even if the authors are unable to move from their current approach or position - then it is important to acknowledge that there are more modern and comprehensive definitions of multimorbidity and that this may have impacted on the findings of the study. Open transparent reporting of this sort is important to allow the field of research to continue to evolve for patient benefit and so that we do not get stuck by citing previous approaches - especially if those have become dated or superseded by a whole body of more modern definitions.

Minor comments

4. At the top of the methods you can say your reported the study as per the RECORD guidelines (which is an extension of STROBE) so you do not need to state both STROBE and RECORD.

5. Could you provide a more specific term than consumer in the newly added PPI section? It would be good to clarify if this was patient input (if so is it someone with lived experience of multimorbidity?) or was this a more general lay/public representative?

VERSION 2 - AUTHOR RESPONSE

Reviewer: 1
Prof. Filipe Prazeres, Universidade da Beira Interior Faculdade de Ciencias da Saude, CINTESIS
Comments to the Author:
Thank you for replying to my questions, nothing else to state.

Thank you for reviewing our manuscript.

Reviewer: 2
Dr. Marlous Hall, University of Leeds
Comments to the Author:
Whilst some elements raised in my review have been addressed or rebutted suitably - there are a few aspects which are still outstanding and I feel these don't quite meet the expected reporting standards. I have also provided some further clarity on my point about the definition of multimorbidity which may have been misinterpreted by the authors in my earlier review - and some further thought on the implications of this in the manuscript would be beneficial.

Thank you for providing these additional points for clarification.

1. Regarding the STROBE/RECORD statement "Bias #9 Describe any efforts to address potential sources of bias". The authors have added "This was to avoid the overrepresentation of frequently hospitalised individuals." (p. 20, line 136-142) alongside their inclusion criteria in the methods - however I do not feel this suitably describes efforts to address potential sources of bias.

As per my original comment - observational research using EHRs are prone to a wide range of biases. It is important, and expected as outlined in the reporting guidelines, to ensure there are efforts made to reduce these biases and that such efforts are reported directly and clearly in the manuscript (usually in methods and discussion). The authors have not expressly listed their thought process on confounder adjustment (or mentioned confounders at all), nor have they mentioned

efforts to reduce other potential sources of bias such as selection bias.

Specifically - the statistical analyses section states that "Multivariate logistic regression was used to determine the association between multimorbidity and frailty on the use of guideline-directed medicines when controlling for the covariates of age, gender, number of regular medicines, the logarithm of serum creatinine on admission and hepatic impairment." The use of the term covariates is a little ambiguous - and it would be better if the authors could be more explicit about whether they consider all the covariates included to be confounders of the exposure/outcome relationship? A brief explanation providing justification for the selection of this particular set of confounders in the methods is also needed, and in the discussion it would be appropriate to address whether there were other potential confounders which may influence the exposure/outcome relationship that were unable to be included (e.g. due to poor data quality or lack of availability of information). For example ethnicity, socioeconomic deprivation, changes in clinical practice or recording over time are a few potential confounders that haven't been mentioned or included. It is important to highlight potential for residual confounding in the findings and highlight that their omission may have resulted in some bias as a study limitation. Incorporating these elements would better address the reporting standards item on bias - which has been designed to improve the scientific integrity of published observational research.

Thank you for this comment. We have added a section to the Methods section explicitly stating the confounders in the exposure/outcome relationship and justified those that have been selected. It reads as follows.

"Multivariate logistic regression was used to determine the association between multimorbidity and frailty with the use of guideline-directed medicines. The confounding factors of age, gender, number of medications, renal function and the presence of chronic liver disease were chosen as these clinically influence medication use. Older age is associated with an increased risk of cardiovascular events and medication-related harm. Gender was chosen to account for sex-based differences in medication use. The number of medications was chosen to represent baseline medication burden which may influence prescribing practices through risk of drug-drug interactions and resulting caution in prescribing. Renal and hepatic function represent the primary modes of drug excretion and influence medication use. Renal function was assessed using the serum creatinine on admission and hepatic function using select ICD-10 codes (see Appendix C). The other potential confounders were socioeconomic status, ethnicity and inpatient blood pressure readings for hypertension treatment. Socioeconomic status and ethnicity were not available in our dataset. Blood pressure readings were not included in the analysis given that these are a dynamic vital sign and that blood pressure targets for hypertension are based on outpatient rather than inpatient measurements. Changes in prescribing patterns over time were not considered a confounder given the study was undertaken over a defined 18-months period and these medications had all been available and recommended by guidelines for several years prior. Given this, the confounders adjusted for in the logistic regression were age, gender, number of regular medicines, the logarithm of serum creatinine on admission and hepatic impairment. (p. 12, lines 221-240)

The line in the Discussion outlining unmeasured confounding as a limitation in the logistic regressions has been expanded to include specific factors that were not included and may have introduced bias. It reads as follows.

“Sixthly, there may be unmeasured confounding in the logistic regressions to determine the factors associated with use of guideline-directed medicines. Factors such as socioeconomic status and ethnicity were not included in the analysis and this may have introduced confounding.” (p. 22-23, lines 471-477)

The elements of the study design that were chosen to minimise biases, including selection bias, have been highlighted in the Methods.

“The inclusion criteria were all adults aged ≥45 years with first admissions via the emergency department under specific units who manage patients with general comorbidities (see Appendix A), such as General Medicine, General Surgery and Geriatrics. These broad pre-specified inclusion criteria were applied to avoid selection bias through the overrepresentation of frequently hospitalised individuals. Patients were required to have a length of in-patient stay greater than 48 hours to allow sufficient time for the treating clinicians to chart their preadmission medications into the EMR. Patients with palliative care consultations and who did not survive to discharge were excluded. It is acknowledged that there will be selection bias introduced by the minimum length of stay, exclusion of patients who did not survive to discharge and exclusion of patients admitted under highly specialised units. However, this was necessary to achieve the study aims whilst ensuring that the cohort remained representative of the general inpatient population.” (p. 8-9, lines 136-152)

The potential sources of bias have also been highlighted in the Limitations.

“Fourthly, the use of ICD-10 codes from hospital coding data may be insensitive for the detection of select chronic medical conditions introducing misclassification bias.“ (p. 22, lines 460-462)

“Sixthly, there may be unmeasured confounding in the logistic regressions to determine the factors associated with use of guideline-directed medicines. Factors such as socioeconomic status and ethnicity were not included in the analysis and this may have introduced confounding. Seventhly, the use of patients who survived to discharge may have introduced collider bias as illness severity may influence both prescribing and survival. This would mean that associations may be found that would not have existed in the complete population. Finally, the generalisability of these findings to specialist hospital units and other settings including regional or private hospitals, community dwelling adults and international health systems is unclear.” (p. 22-23, lines 471-477)

2. Very minor comment on the same sentence - should this instead read "the association between multimorbidity and frailty *with* the use of guideline-directed

medicines..."?

This has now been corrected as suggested and now reads as follows.

"Multivariate logistic regression was used to determine the association between multimorbidity and frailty with the use of guideline-directed medicines when controlling for the covariates of age, gender, number of regular medicines, the logarithm of serum creatinine on admission and hepatic impairment." (p. 12, line 221-222)

3. Multimorbidity definition. I wanted to clarify with the authors that I was not disputing the WHO's definition of multimorbidity being the co-existence of two or more long term conditions and apologise if the way I phrased my review caused confusion. Rather I want to highlight that the way that this definition of 2+LTCs is applied or implemented in research can cause problems. My concern is two-fold 1) No two LTCs are the same yet they all are aggregated in the same way with the same weight - there is a huge loss of valuable information here by reducing this to a binary concept which does not account for the specific conditions individuals have. However - I note the authors have now added a statement to acknowledge this limitation of their approach to address this point.

Thank you for this additional clarification and acknowledging that we have added this as a limitation of multimorbidity definition used in this study.

2) Another concern is the heterogeneity in the number of LTCs included in the count of 2+LTCs in research and alongside that a lack of transparency of how and why the choice of LTCs has been arrived at - this element has not yet been addressed by the authors. The paper I cited in my initial review highlights very clearly that the prevalence and impact of 2+LTCs is heavily influenced by the specific conditions included (e.g. one paper might include a limited set of conditions, another might comprehensively include LTCs relating to all body systems). In this study - the authors have cited the Barrett et al paper from 2012 to inform their choice of LTCs to include. This was a highly influential paper - laying the foundations for much subsequent multimorbidity research. However it would be good for the authors to justify their choice of these conditions more strongly - given the field of research has significantly advanced in recent years and there have been many subsequent papers reviewing the definitions of multimorbidity through delphi processes aiming to reach a consensus (e.g. one example, but not the only one, is the study by Ho et al). I very much appreciate that there remain multiple ways to define multimorbidity despite the efforts of previous consensus studies - but this is all the more reason to provide a discussion and clear justification for the choice of included LTCs included in this study. Even if the authors are unable to move from their current approach or position - then it is important to acknowledge that there are more modern and comprehensive definitions of multimorbidity and that this may have impacted on the findings of the study. Open transparent reporting of this sort is important to allow the field of research to continue to evolve for patient benefit and so that we do not get stuck by citing previous approaches - especially if those have become dated or superseded by a whole body of more modern definitions.

Thank you for this comment on the evolution of the multimorbidity definitions since this study was devised. The Methods section has been updated to include a justification of the use of the Barnett et al. (2012) as well as to indicate that Rx-Risk V pharmaceutical comorbidity index was used as a sensitivity analysis.

"Multimorbidity was identified using a published count of 40 chronic medical conditions from hospital coding data[27]. Individuals with two or more coded chronic medical conditions during the hospital admission were considered to have multimorbidity in accordance with the previous study[27]. This definition was chosen as the most widely used and internationally adopted method of identifying multimorbidity from administrative data. It is one of the recommended methods when measuring prevalence and had published ICD-10 code lists when this study was devised.[28] Given the potential for undercoding of chronic medical conditions in administrative data, a sensitivity analysis was performed by identifying multimorbidity using the Rx-Risk V comorbidity index based on medications orders." (p. 9-10, lines 171-181)

Both estimates of multimorbidity using Barnett et al. (2012) and Rx-Risk V comorbidity index have been included in the Discussion and Conclusions. There is a line indicating that there is a study in the Australian setting showing that RxRisk is likely to be more sensitive than hospital coding data. This has been edited slightly for clarify.

"The RxRisk comorbidity score has been shown to have fair agreement with self-reported multimorbidity and is likely to be more sensitive than hospital coding data in the Australian setting[1]." (page 18, line 158-160)

As suggested, the use of the Barnett et al. definition using ICD-10 codes has been listed as a limitation that may have underestimated the prevalence of multimorbidity. It has also been acknowledged that there are now more modern and comprehensive code lists that has been published.

"Since this study was devised, more modern and comprehensive consensus definitions of multimorbidity including revised ICD-10 code lists have been published.[56 57] Although these largely overlap with the chronic diseases included in the Barnett et al. list, they include some additional conditions and may mean that the prevalence of multimorbidity based coding data was underestimated in this study." (page 22, line 452-456)

Minor comments

4. At the top of the methods you can say your reported the study as per the RECORD guidelines (which is an extension of STROBE) so you do not need to state both STROBE and RECORD.

This sentence has been revised to reference the RECORD guideline.

"A cross-sectional study reported in accordance with the RECORD statement[22] was conducted using hospital admission data from the Royal Adelaide Hospital and the Queen Elizabeth Hospital in Adelaide, South Australia, between January 2022 and June 2023." (p. 8 line 126-128)

5. Could you provide a more specific term than consumer in the newly added PPI

section? It would be good to clarify if this was patient input (if so is it someone with lived experience of multimorbidity?) or was this a more general lay/public representative?

This sentence has been revised to provide further context on the consumers' experience of multimorbidity.
"A consumer with lived experience as a carer of a family member with multimorbidity provided feedback on the results, their interpretation and the implications for patients. (p. 13 line 259-260)

Thank you for your consideration of this revised manuscript.

VERSION 3 - REVIEW

**Reviewer**	**2**
**Name**	**Hall, Marlous**
**Affiliation**	**University of Leeds**
**Date**	**19-Dec-2025**
**COI**	

Many thanks to the authors for carefully considering and responding to the final set of considerations I had regarding the manuscript. These have now been fully addressed in the revised manuscript and the paper is now well aligned with reporting standards.
